# Supplementary material for: Solvent-Free Fabrication of Thick Electrodes in Thermoplastic Binders for High Energy Density Lithium-Ion Batteries
Source: Nanomaterials (Basel). 2022 Sep 23;12(19):3320. doi: 10.3390/nano12193320 (PMC9565545; doi:10.3390/nano12193320)
Supplement: Supplementary file 1 [file nanomaterials-12-03320-s001.zip › nanomaterials-1891260-supplementary.pdf]

nanomaterials

MDPI

Supplementary Materials

Solvent-Free Fabrication of Thick Electrodes in Thermoplastic Binders for High Energy Density Lithium-Ion Batteries

Han-Min Kim [1,2], Byeong-Il Yoo [1,2], Jin-Woo Yi [1], Min-Jae Choi [2,*] and Jung-Keun Yoo [1,3,*]

1 Composites Research Division, Carbon Composites Department, Korea Institute of Materials Science (KIMS), Changwon 51508, Korea
2 Department of Chemical and Biochemical Engineering, Dongguk University, Seoul 04620, Korea
3 Advanced Materials Engineering Division, University of Science and Technology (UST), Daejeon 34113, Korea
* Correspondence: minjae.choi@dgu.ac.kr (M.-J.C.); yoojk@kims.re.kr (J.-K.Y.)

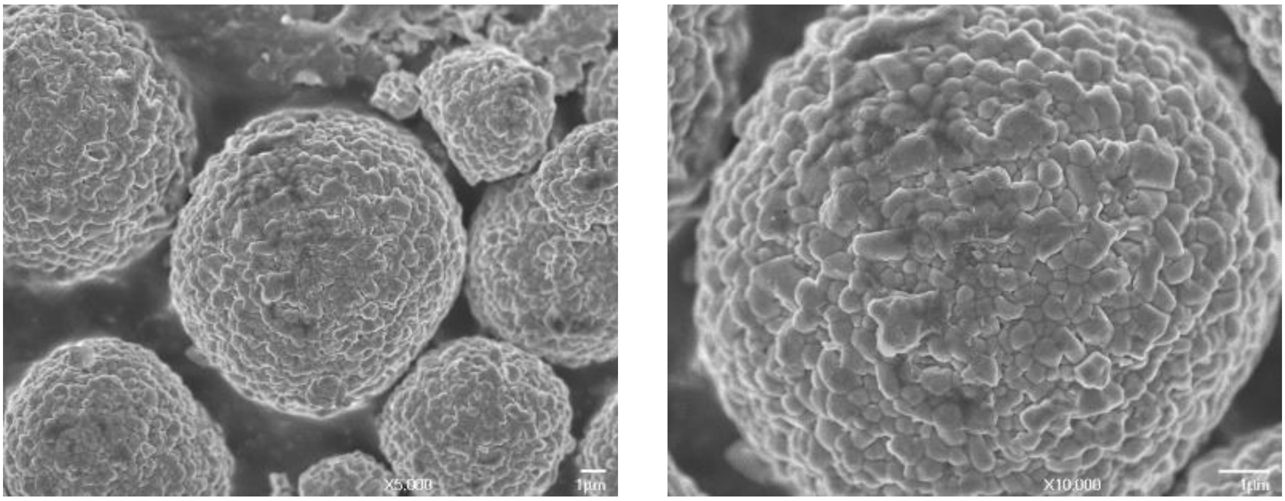

Figure S1. SEM image of NCM811 surface. Scale bar: 1μm.

Table S1. NCM811 particle analysis information.

NCM 811	Particle-size Distribution (μm)			Specific Surface Area (m^2/g)	Excess Lithium (%)		
	**Dmin**	**D50**	**Dmax**		**LiOH**	**Li_2CO_3**	**Total**
	0.7	11.3	28.9	0.46	0.269	0.140	0.555

Table S2. Electrode thickness change before/after wetting the electrolyte.

		**Wet PVDF**	**Dry PVDF**	**Wet Phenoxy Resin**	**Dry Phenoxy Resin**
Thickness (μm)	Before wetting	150.48	152.47	149.75	150.63
	After wetting	164.02	167.72	155.74	155.15
Thickness Change (%)		108	110	104	103

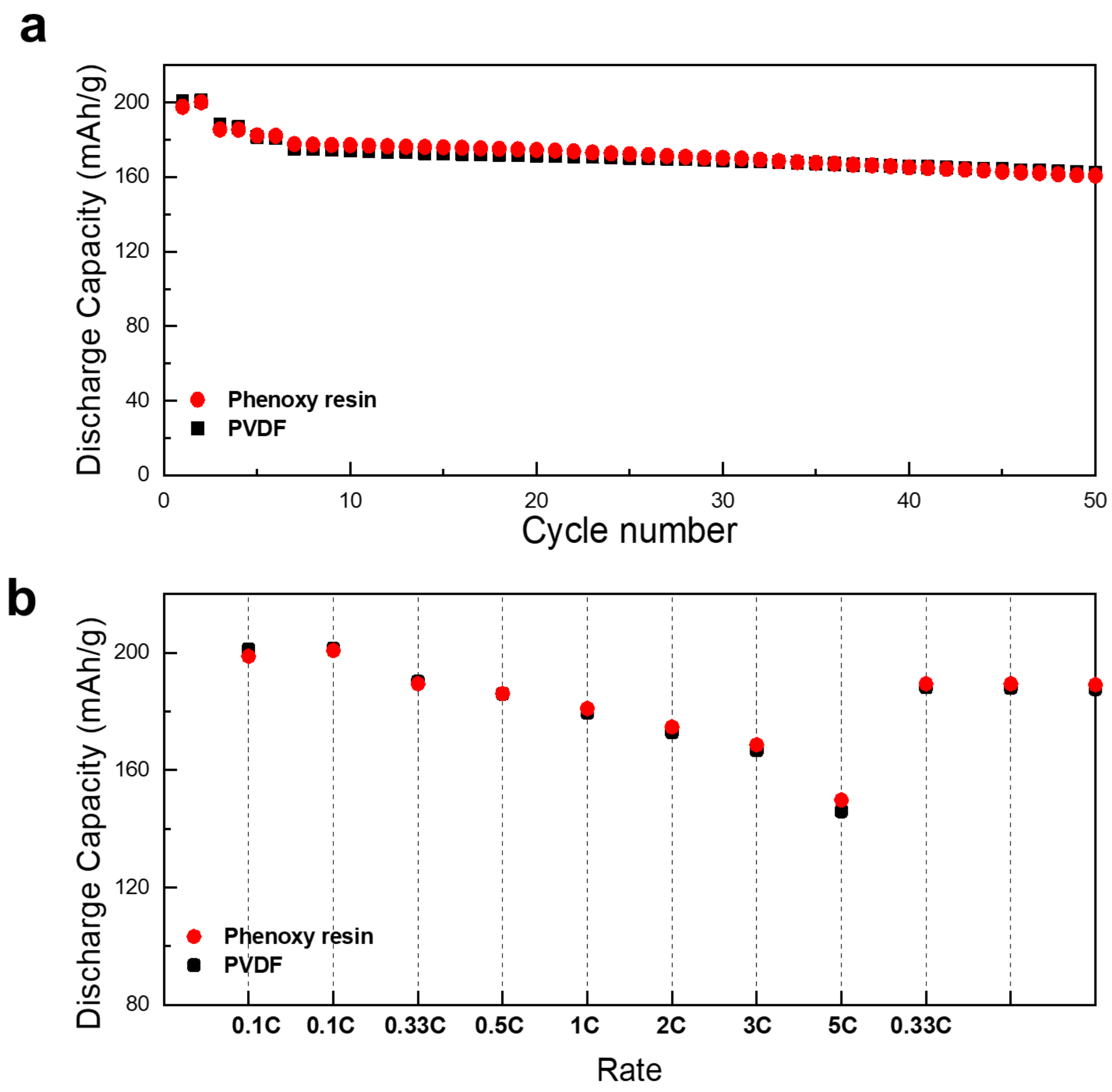

Figure S2. Characterizations of electrochemical properties of Loading ~10 mg/cm^2: Cycle performances of Phenoxy resin and PVDF at 1C. (**a**) Rate performances of Phenoxy resin and PVDF at various current density of Phenoxy resin and PVDF. (**b**).

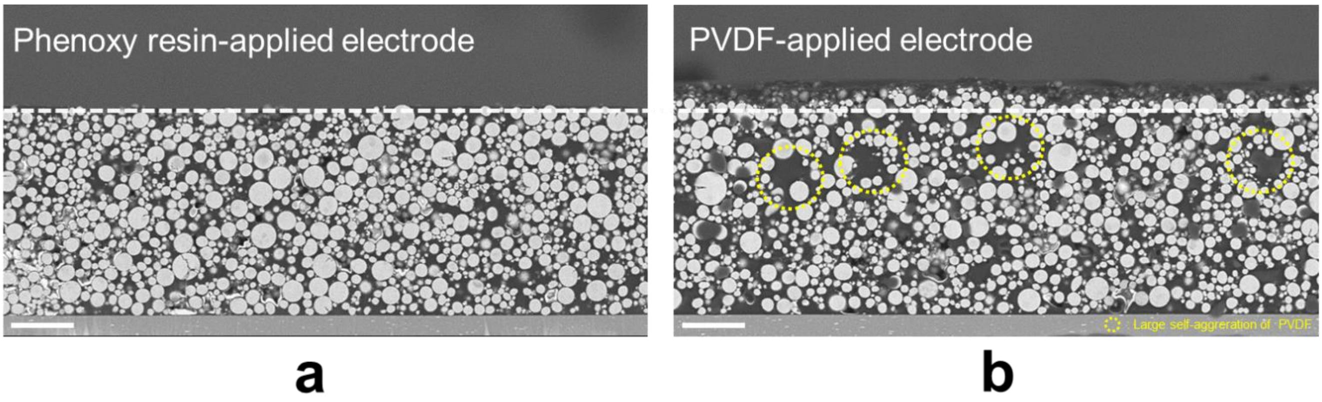

Figure S3. Cross-sectional SEM image of electrode surface using (**a**) Phenoxy resin (**b**) PVDF. Scale bar = 50 μm.

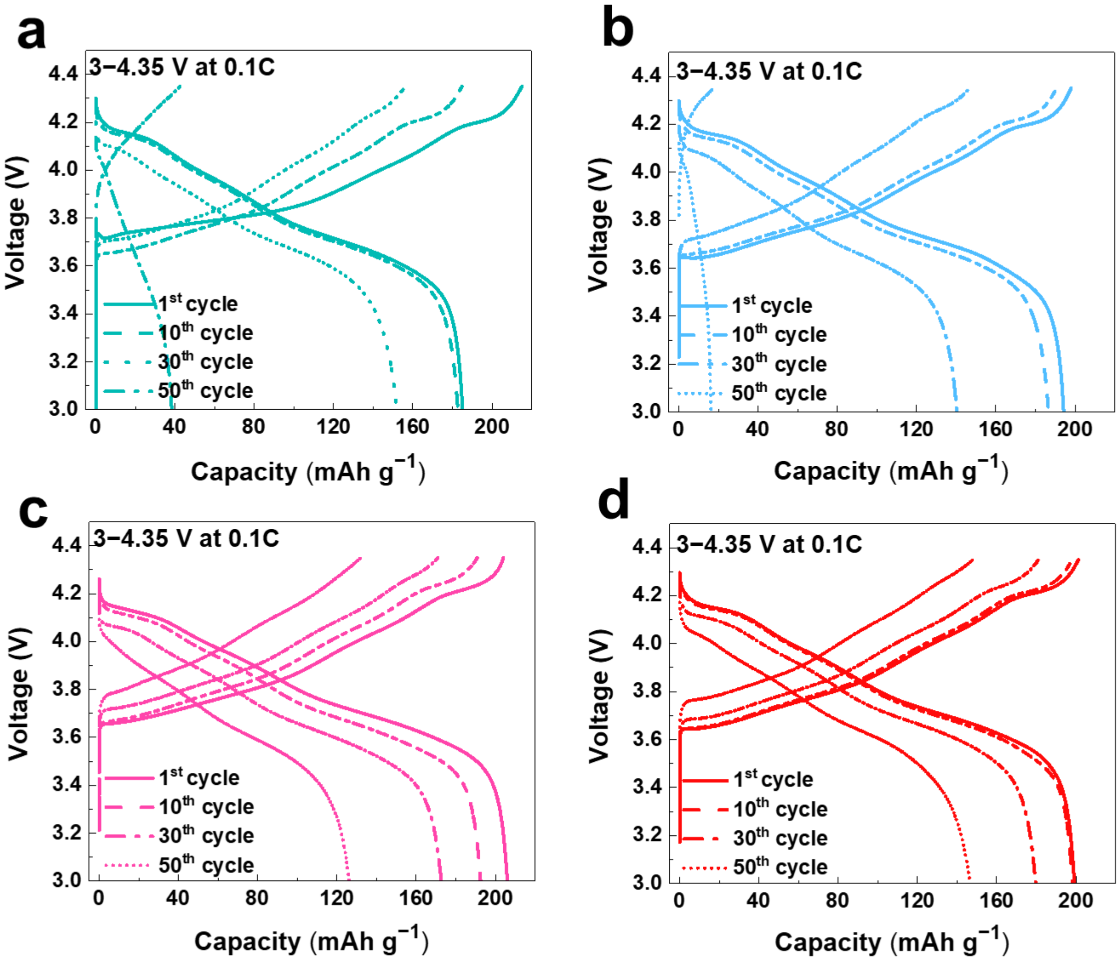

Figure S4. Charge/discharge curves at 1st, 10th,30th and 50th cycles of (**a**) Wet PVDF (**b**) Dry PVDF (**c**) Wet Phenoxy resin (**d**) Dry Phenoxy resin.

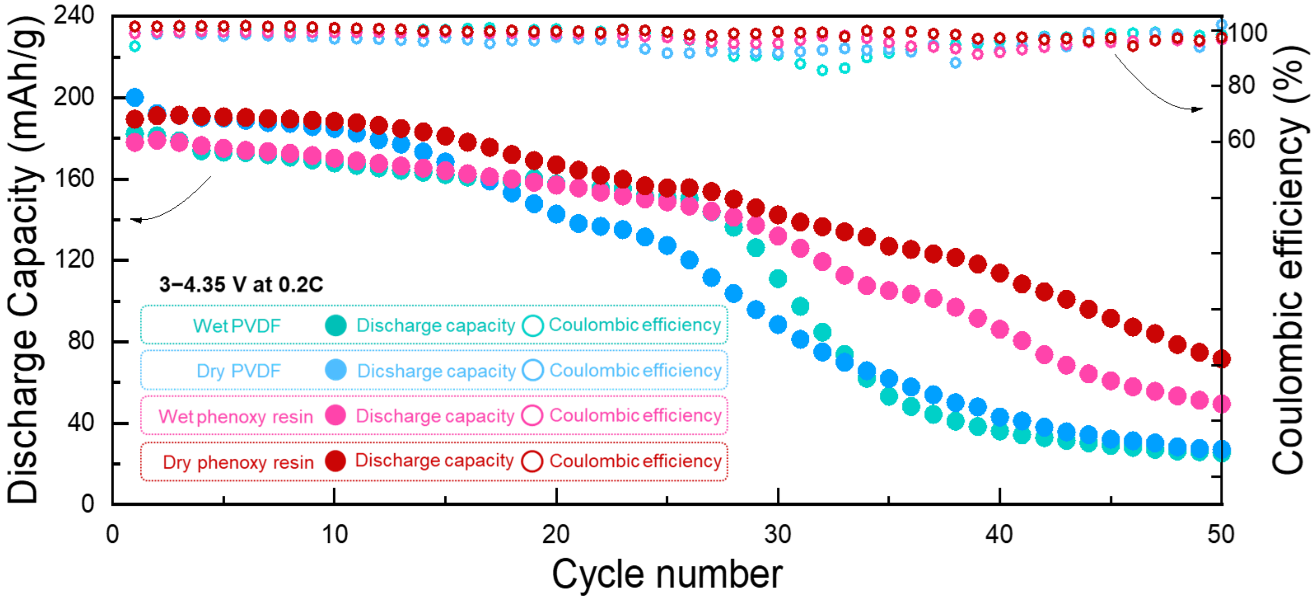

Figure S5. Cycle performances of Wet PVDF, Dry PVDF, Wet Phenoxy resin, Dry Phenoxy resin at 0.2C.
